# Safety, effectiveness, and impact on quality of life of self-administration with plasma-derived nanofiltered C1 inhibitor (Berinert®) in patients with hereditary angioedema: the SABHA study

**DOI:** 10.1186/s13023-018-0797-3

**Published:** 2018-04-10

**Authors:** Andrea Zanichelli, Giulia Maria Azin, Federico Cristina, Romualdo Vacchini, Teresa Caballero

**Affiliations:** 1 Department of Biomedical and Clinical Sciences Luigi Sacco, University of Milan, ASST Fatebenefratelli Sacco, Milan, Italy; 2 Allergy Department, Hospital La Paz Institute for Health Research, Madrid, Spain

**Keywords:** Hereditary angioedema, C1 esterase inhibitor, Self-administration, Survey, Quality of life, TSQM

## Abstract

**Background:**

Hereditary angioedema with C1 inhibitor deficiency is a disabling, potentially fatal condition characterized by recurrent episodes of swelling. Self-treatment is recommended, in order to reduce admissions to the Emergency Room and the time between the onset of the attack and the treatment, resulting in a better treatment outcome and an improved quality of life (QoL). The purpose of this study is to assess the safety, tolerability, and effect on QoL of self-administration of pnf C1-INH for IV use (Berinert®).

**Methods:**

An observational, monocenter, prospective study was designed.

Patients referring to a center for angioedema that attended two sessions of self-infusion training course in the period March 2014–July 2015 were enrolled in the study. The primary endpoint was to monitor the safety and feasibility of pnf C1-INH self-infusion. The secondary endpoint was to evaluate the effect of self-infusion on the QoL, by means of the HAE-QoL questionnaire and the need for access to Emergency Room for infusion of Berinert®. Patients’ medical history data were collected upon the first visit and questionnaires were filled after each attack treated with Berinert® (diary and *Treatment Satisfaction Questionnaire for Medication*) and upon the first visit and the follow-ups (HAE-QoL).

**Results:**

Twenty patients were enrolled (median age = 42, IQR: 39–49; 60% females). Fifteen patients completed the study. A total of 189 attacks were recorded (annual median rate of 4 attacks/patient). Patients waited a median of 2 h (IQR: 1–4) before self-administration, and the resolution of the attack occurred after a median of 6 h (IQR: 4–11). Most attacks were abdominal (39%) and peripheral (22%). 92% of the attacks were treated through self−/caregiver-administration. In most attacks no side effects were reported. The number of attacks with side effects decreased over time, from 37% to 13%. Global satisfaction grew over time during the study period, reaching statistical significance over the first 6 months. The median total HAE-QoL score at baseline was 86 (IQR: 76–103) and improved in a non-significant manner throughout the study period. 8% of the attacks treated with Berinert® required ER admission/healthcare professional help in the study period, compared with 100% in the 3 years before enrollment (*p* < 0.0001).

**Conclusions:**

Self-administration of pnf C1-INH is safe, and increases patients’ confidence in the treatment, showing also a trend towards an improvement in QoL. It reduces the need for ER admission/healthcare professionals help for the acute attacks, as well as the related costs.

## Background

Hereditary angioedema with C1 inhibitor deficiency (C1-INH-HAE) is a rare autosomal dominant disease featuring the occurrence of angioedema attacks in several body sites, in particular extremities, genitourinary tract, face, oropharynx, larynx, and abdomen [1]. The phenotypical manifestations are due to mutations in the *SERPING1* gene, encoding C1-INH [2].

Data on the prevalence of C1-INH-HAE are not univocal, but all agree with the absence of racial or gender preference. A nationwide survey conducted in Italy in 2015 found a prevalence equivalent to 1:64,935 [3]. Accounting also for undiagnosed and misdiagnosed patients, the real prevalence may be around 1:50,000, as generally reported in literature.

In type I C1-INH-HAE, reduced plasma levels of C1-INH can be detected, whereas in type II C1-INH-HAE the mutated gene produces a dysfunctional protein [1]. In both cases, the reduced amount of functional C1-INH is responsible for the excessive local generation of bradykinin, which in turn results in increased vascular permeability, subsequent leakage of plasma from the capillaries in the deep layers of the skin or the mucosae, and angioedema formation. Patients affected by C1-INH-HAE suffer from recurrent attacks, with variable frequency, which can change body location inter- and intra-attack, last up to 5 days, and be life-threatening, if occurring at larynx level (asphyxia). Abdominal attacks may result in symptoms similar to those observed during intestinal occlusion syndrome, sometimes associated with ascites and hypovolemic shock [4]. Therefore, C1-INH-HAE is a debilitating disease, with serious effect on the quality of life (QoL) [5]. International consensus documents recommend that patients with C1-INH-HAE experiencing an attack, irrespective of its location, should be treated at the earliest convenience with specific-disease treatments [6–9]. Three active medications are licensed for the treatment of acute attacks [10]:

- C1-INH concentrate;
- bradykinin B2 receptor (BK-B2R) antagonist (icatibant);
- kallikrein inhibitor (ecallantide) (licensed in the US only).

All treatments are thought to target the bradykinin pathway and to aim at the reduction of bradykinin production (C1-INH concentrate [11] and ecallantide [12]) or at the inhibition of bradykinin activity, through the binding to its receptor (icatibant [13]).

C1-INH is available as plasma-derived nanofiltered (pnf) or recombinant human (rh) concentrate.

A practical limitation of the C1-INH replacement therapy is the need for intravenous (IV) administration. Patients usually receive treatment for acute attacks at a clinic or hospital, but any delay in accessing a care facility can impact the treatment outcome [2]. Home-based treatment and self-treatment can help reduce the time between the onset of the attack and the start of treatment, thus preventing any further progress of the attack, reducing the severity of the attacks, and resulting in an improved quality of life [2, 14–16]. Self-administration is also recommended by international guidelines [6, 9].

Since 2011, Berinert® (CSL-Behring, Marburg, Germany) has been approved for self-administration. Self-infusion of pnf C1-INH is feasible, after a suitable training under the supervision of health care providers (HCPs).

The purpose of this study is to monitor the implementation of the self-administered treatment with pnf C1-INH (Berinert®) in the routine clinical practice of a referring center for C1-INH-HAE, and to assess the safety, tolerability, and effect on QoL of the IV self-administration of pnf C1-INH.

## Methods

### Design of the study

***S****elf-****A****dministration with pnf C1-INH (****B****erinert®) in patients with* ***H****ereditary* ***A****ngioedema* (SABHA) is an observational, monocenter, prospective study. The primary endpoint is monitoring the safety and feasibility of pnf C1-INH self-infusion in a “real-world” setting. The secondary endpoint is to evaluate the effect of self-infusion on the QoL and the need for access to Emergency Room for infusion of Berinert®. C1-INH-HAE patients referring to the Luigi Sacco Hospital, in Milan, that attended two sessions of self-infusion training course in the period March 2014–July 2015 were enrolled in the study.

### Self-administration training

The training course for self-administration of pnf C1-INH consisted of a theoretical session on the storage, preparation, and intravenous administration of the drug, followed by a practical session in which patients could practice making intravenous infusions on a fake arm and on themselves. Several administration courses were performed with small groups of patients and caregivers (maximum 10 people). Patients were given the opportunity to attend a second course of self-infusion technique. If patients were not feeling confident with self-administration, they also had the opportunity to perform their first self-infusion at our Center, under the supervision of healthcare professionals.

### Patients

Patients followed-up at our center were contacted by phone and were offered to participate in the study if they had at least one attack/year and had been previously treated with Berinert®.

Patients included in the study were ≥ 18 years, had a clinical and laboratory diagnosis of type I or type II C1-INH-HAE, were able and willing to comply with the requirements of the study protocol, and had a documented attendance to a C1-INH concentrate self-infusion training course. In some cases, caregivers attended the training together with the patients: in the analysis, any drug administration performed by caregivers is considered as self-administration, because there was neither the need of a physician’s/nurse’s help at home, nor any need for admission to the Emergency Room (ER).

The diagnosis of C1-INH-HAE was based on a family and/or personal history of recurrent angioedema without urticaria and on C1-INH antigenic and/or functional plasma levels below 50% of normal. Patients were diagnosed with type I C1-INH-HAE when functional and antigenic C1-INH were ≤ 50% of normal, and with type II C1-INH-HAE when functional C1-INH was ≤50% and antigenic C1-INH was > 50% of normal.

Patients were excluded if judged incapable of managing the IV self-infusion, if affected by mental conditions making them incapable of understanding the nature, objectives and possible consequences of the study, or in the presence – in the investigator’s opinion – of any other condition which could possibly interfere with the patient’s participation (concomitant diseases, uncooperative attitude, inability to return for the follow-up).

### Assessments and data collection

Assessment visits at the clinic were planned as follows: screening/enrollment (visit 1), month 3 (visit 2), month 6 (visit 3), and month 12 (visit 4, end of study).

Patients had to complete two surveys after each attack treated with Berinert®:

- the diary reporting the characteristics of the attack (duration, severity, outcome) and the feasibility of the pnf C1-INH (Berinert®) infusion;
- the *Treatment Satisfaction Questionnaire for Medication* (TSQM), version 1.4 [17, 18], which collects information about effectiveness (3 items), side effects (4 items), convenience (3 items), and global satisfaction (3 items) about the drug used, in this case pnf C1-INH (Berinert®), returning scores ranging from 0 to 100 for each item.

In addition, patients were asked to fill in a questionnaire on the quality of life (HAE-QoL, 6 months recall Italian version [19]) three times during the study (visit 1, 3, and 4). The HAE-QoL questionnaire assesses physical functioning and health (4 items, score ranging from 4 to 23), disease related stigma (3 items, score ranging from 3 to 15), emotional role and social functioning (4 items, score ranging from 4 to 20), concern about offspring (2 items, score ranging from 2 to 10), perceived control over illness (4 items, score ranging from 4 to 20), mental health (4 items, score ranging from 4 to 24), and treatment difficulties (4 items, score ranging from 4 to 23).

At the enrollment visit, the following data were collected for each patient: date of birth, gender, race, medical history, concomitant treatment, date of diagnosis, type of C1-INH-HAE, functional C1-INH levels at diagnosis, treatments used by patients for acute attacks, prophylaxis therapy with androgens or antifibrinolytics, and retrospective data about the economic impact on public health (admission to ER/Intensive Care Unit [ICU], intubations). In case of concerns about the data collected, conflicts were resolved by consulting hospital records and calling patients by phone. All patients gave their informed consent, completed the baseline HAE-QoL, and were trained in the self-administration of pnf C1-INH (Berinert®).

In the period from the enrollment to the end of the study, 12 months later, in case of attacks subjects could self-administer pnf C1-INH (Berinert®) at home, following the instructions on preparation, handling, and administration; if the attack was potentially life-threatening, or if the subject did not feel comfortable with the self-infusion for any reason, the administration could be carried out at the clinic by a health care professional, according to normal clinical practice. When the self-treated attack was resolved, the patient made an entry in the diary and filled in the TSQM.

During follow-up visits, subjects provided the diary and the filled out TSQM, which were reviewed by the Investigator for completeness: any discrepant/unclear information was reconciled. For each patient, the following data were collected: attacks treated with self-administration (date, site, severity, and outcome), compliance, concomitant treatment, and adverse events.

Follow-up visits were planned irrespective of any subsequent home-treated HAE attacks.

### Ethics, consent and permissions

An institutional approval was issued by the hospital Ethics Committee, with protocol number 110/2013/80/2012/AP. All patients enrolled in this study gave their informed consent to use their anonymized data.

### Statistical analyses

Continuous variables were reported as median and interquartile range (IQR), while categorical variables were reported as absolute frequencies and percentages.

The differences in QoL recorded at visit 1, 3, and 4 were evaluated through Welch’s t-test in the case of normally distributed variables (Shapiro-Wilk test to check for normality and Fisher’s exact test for the differences among the variances), or through the Wilcoxon test, in the case of non-normally distributed variables.

The processing of the results and the calculation of the descriptive statistics (median, IQR, etc.) was performed in MS Excel 2013®. Inferential analyses (statistical hypothesis, *p*-value calculation, etc.) were performed by means of the R software, version 3.1.2 [20].

## Results

### Patients included and clinical history

Twenty out of 34 patients that were reached by phone were included in the study. Data from these patients were collected and analyzed. Personal data, clinical history, and resource consumption over the previous years were available for all patients. After visit 1, four patients were excluded from the analysis of the diary, TSQM, and HAE-QoL: 1 patient moved abroad and 3 patients were lost at the first follow-up. In addition, one patient had no attacks during the study period.

Table 1 shows the patients’ characteristics at visit 1 (*n* = 20).

**Table 1:** Characteristics of the enrolled population

Patients analyzed at visit 1	*N* = 20
Age (median, IQR) (years)	42 (39–49)
Gender (female)	12 (60%)
Race (Caucasian)	19 (95%)
Age at HAE diagnosis (median, IQR) (years)	18.5 (13–32)
HAE etiology (% type 1)	20 (100%)
Patients under prophylactic treatment (total)	6 (30%)
• Androgens	5 (83.3%)
• Antifibrinolytics	1 (16.7%)
• Pnf C1-INH	0 (0%)
Treatment used by patients for acute attacks before the study	
• Pnf C1-INH	18 (90%)
• Icatibant	12 (60%)

Median age at enrollment was 42, and 60% of the subjects were female. One patient lost at follow-up was South-American. Median age at the HAE diagnosis was 18.5. All patients enrolled had type 1 HAE.

Six out of 20 patients (30%) were on prophylactic therapy at the enrollment visit: 5 patients were taking androgens (among them, 2 were lost at follow-up) and 1 patient was on antifibrinolytics.

Before being enrolled in the study, patients used pnf C1-INH (18 out of 20, 90%) or icatibant (12 out of 20, 60%) as a treatment for acute attacks. No patients had ever used rh C1-INH.

Among the most common concomitant pathologies ongoing upon enrollment, 7 patients (35%) had cardiovascular diseases, 3 patients (15%) had ENT diseases, and 3 patients (15%) had gastrointestinal diseases.

The group (*n* = 15) as a whole recorded a total of 388 attacks treated with Berinert® in the 3 years before enrollment (that means an average of 129 attacks/year and amedian of 3 attacks/year per patient, IQR: 1–10). Data about the site and severity of the attacks were available for 11 patients, who, as a group, had an annual mean of 112 attacks. Most attacks were abdominal (45%) and peripheral (44%). The others were categorized as genitourinary (10%) and orofacial (1%). 64% of the attacks resulted in mild to moderate symptoms.

### Characteristics of the attacks and adverse events of pnf C1-INH self-administration

During the study period, a total of 189 HAE attacks treated with Berinert® were recorded, corresponding to an annual median rate of 4 attacks/patient (IQR: 1–16).

Patients waited a median of 2 h (IQR: 1–4) before performing the self-administration, and the complete resolution of the attacks occurred after a median of 6 h (IQR: 4–11). Most attacks were abdominal (39%) and peripheral (22%). Orofacial attacks were 3%, laryngeal 2%, and genitourinary 2%. Other sites accounted for 16% of attacks. Multiple sites attacks, mainly abdominal and cutaneous, were reported by patients in 17% of the attacks. 81% of the attacks resulted in mild to moderate symptoms. The most commonly used infusion site (information available for 85 attacks) was the arm (88%), followed by the hand (11%) and the forearm (1%).

As reported in Table 2, swelling, reddening, burning, and pain were present in about one-third of the infusions. These infusion-related symptoms were mostly mild to moderate. Mild to moderate itch and a feeling of warmth were present only in 1–2% of the attacks.

**Table 2:** Infusion-related signs/symptoms reported by patients at the infusion site during the attacks

Infusion-related signs/symptoms at the infusion site	Absent	Mild	Moderate	Severe
Swelling	123 (66%)	16 (9%)	15 (8%)	33 (18%)
Reddening	122 (65%)	8 (4%)	57 (30%)	0 (0%)
Burning	120 (64%)	47 (25%)	20 (11%)	0 (0%)
Itch	183 (98%)	2 (1%)	2 (1%)	0 (0%)
Feeling of warmth	185 (99%)	2 (1%)	0 (0%)	0 (0%)
Pain	111 (59%)	5 (3%)	56 (30%)	15 (8%)

### Quality of life

The median HAE-QoL total score at baseline was 86 (IQR: 76–103). During the study period, the score increased, which means an increase in HRQoL, but this difference was not statistically significant. Regarding HAE-QoL domains, the greatest increases after 6 months were observed on concern about offspring, perceived control over illness, and mental health, all showing a stable trend between the third and the last visit (Table 3).

**Table 3:** HAE-QoL scores and variations

HAE-QoL score	Basal		After 6 months (visit 3)		After 12 months (visit 4)	
Total (*N* = 15)	88.6 ± 24.4 (75─106)		95.1 ± 24 (85─103)		94 ± 27.6 (86─113)	
Physical functioning and health	15.1 ± 4 (12─16)		15.8 ± 4.7 (15─19)		16 ± 4 (15─20)	
Disease related stigma	10.7 ± 3.5 (8─14)		10.7 ± 4 (8─15)		10.7 ± 3.9 (9─14)	
Emotional role and social functioning	14.4 ± 3.4 (14─16)		14.4 ± 4.4 (14─17)		14.9 ± 4.5 (15─18)	
Concern about offspring	6.2 ± 3.1 (3─9)		7.6 ± 2.6 (6─10)		7.1 ± 3.1 (4─10)	
Perceived control over illness	11.3 ± 4 (9─15)		12.8 ± 3.9 (11─14)		13.1 ± 4.1 (11─16)	
Mental health	14.7 ± 5.4 (11─20)		16.5 ± 5.8 (14─21)		15.2 ± 5.2 (12─18)	
Treatment difficulties	16.3 ± 5.3 (15─20)		17.2 ± 5.4 (15─22)		16.9 ± 5.5 (14─22)	
Differences in HAE-QoL score between the visits	Visit 3 vs 1	*p*	Visit 4 vs 1	*p*	Visit 4 vs 3	*p*
Total (*N* = 15)	6.4 ± 13.5 (4─14)	0.49	5.4 ± 21.4 (−4─15)	0.59	−1.1 ± 13.4 (−6─3)	0.91
Physical functioning and health	0.7 ± 2.8 (−1─3)	0.67	0.9 ± 3 (−1─4)	0.55	0.2 ± 2.2 (0─1)	0.90
Disease related stigma	0 ± 2.3 (−1─1)	1	0 ± 2.8 (−1─1)	1	0 ± 1.4 (−1─0)	1
Emotional role and social functioning	0.1 ± 2.7 (−1─2)	0.71^a^	0.5 ± 2.7 (−1─2)	0.36^a^	0.4 ± 1.6 (−1─2)	0.69^a^
Concern about offspring	1.4 ± 2.3 (0─1)	0.20^a^	0.9 ± 3.1 (0─1)	0.34^a^	−0.5 ± 2 (0─0)	0.87^a^
Perceived control over illness	1.5 ± 2.9 (0─4)	0.32	1.9 ± 3.9 (−1─4)	0.24	0.4 ± 2.9 (− 1─3)	0.82
Mental health	1.8 ± 3.9 (1─4)	0.41	0.5 ± 4.7 (− 2─4)	0.80	−1.3 ± 4.4 (− 2─0)	0.54
Treatment difficulties	0.9 ± 4 (0─3)	0.65	0.6 ± 5.8 (0─2)	0.75	−0.3 ± 4.6 (−1─0)	0.89

HAE-QoL scores and variations were elaborated from the analysis of the questionnaires administered at visits 1, 3 and 4

*p*: *p*-value; ^a^Test di Wilcoxon (violation of the assumption of normality)

**Fig. 1:**
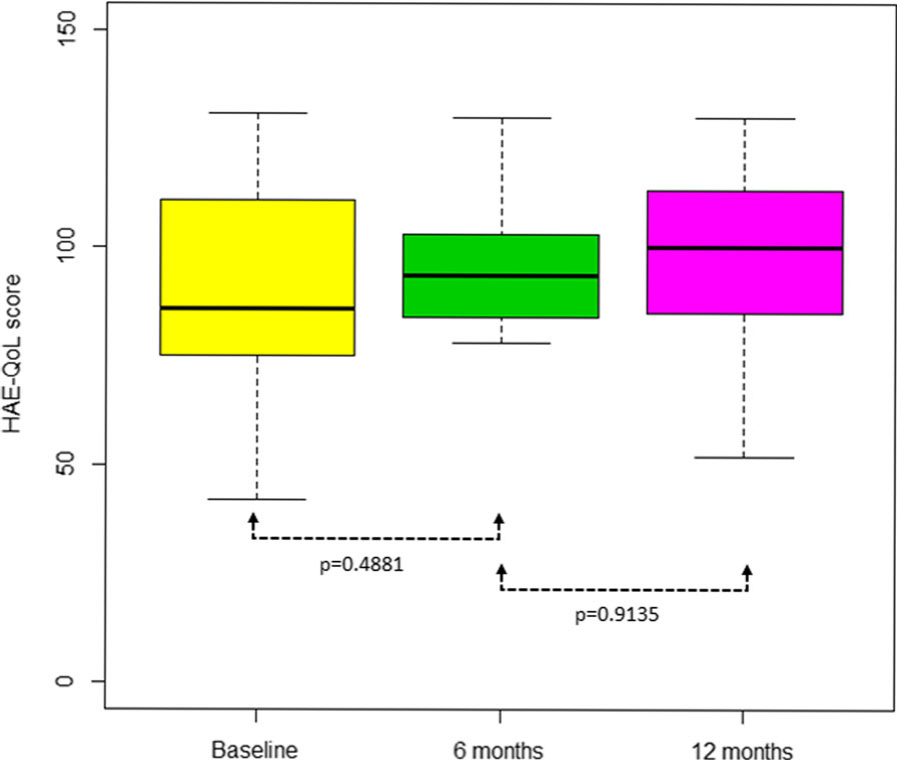
Box plot showing the results of HAE-QoL surveys (medians are in bold)

Figure 1 reports HAE-QoL total scores at different time points. A trend – although not statistically significant – toward an improvement in the quality of life throughout all the study period was reported.

### Patients’ evaluation of the use of pnf C1-INH

Data on the evaluation of pnf C1-INH self-infusion were collected both from the diary and the TSQM. The analysis of the diaries showed that the preparation of the drug before self-infusion was not considered troublesome. 52% of patients reported a high degree of stress related to the self-administration of the drug at visit 1 and visit 2; at the last visit, the number of patients that reported a high degree of stress had decreased to 27%. In most cases the drug was not considered difficult to administer. The drug effect with regard to the resolution of the symptoms was considered positive or very positive in 97% of the attacks. Self-infusion was preferred to hospital treatment in more than 80% of cases, and this preference increased over time.

The results of the TSQM surveys are summarized in Table 4.

**Table 4:** Results of the TSQM surveys

Items	Visit 2 (*n* attacks = 43)	Visit 3 (*n* attacks = 62)	Visit 4 (*n* attacks = 84)	Total (*n* attacks = 189)
Effectiveness (median; IQR)	67 (56–69)	56 (50–72)	67 (50–79)	67 (50–78)
Side effects (median; IQR)	100 (63–100)	100 (100–100)	100 (100–100)	100 (100–100)
• Number of attacks with side effects	16 (37%)	15 (24%)	11 (13%)	42 (22%)
Convenience (median; IQR)	50 (44–56)	50 (44–54)	50 (44–50)	50 (44–56)
Global satisfaction (median; IQR)	53 (46–69)	63 (61–69)	73 (53–85)	63 (53–83)

Scores range from 0 (extremely negative) to 100 (extremely positive) for each item

**Fig. 2:**
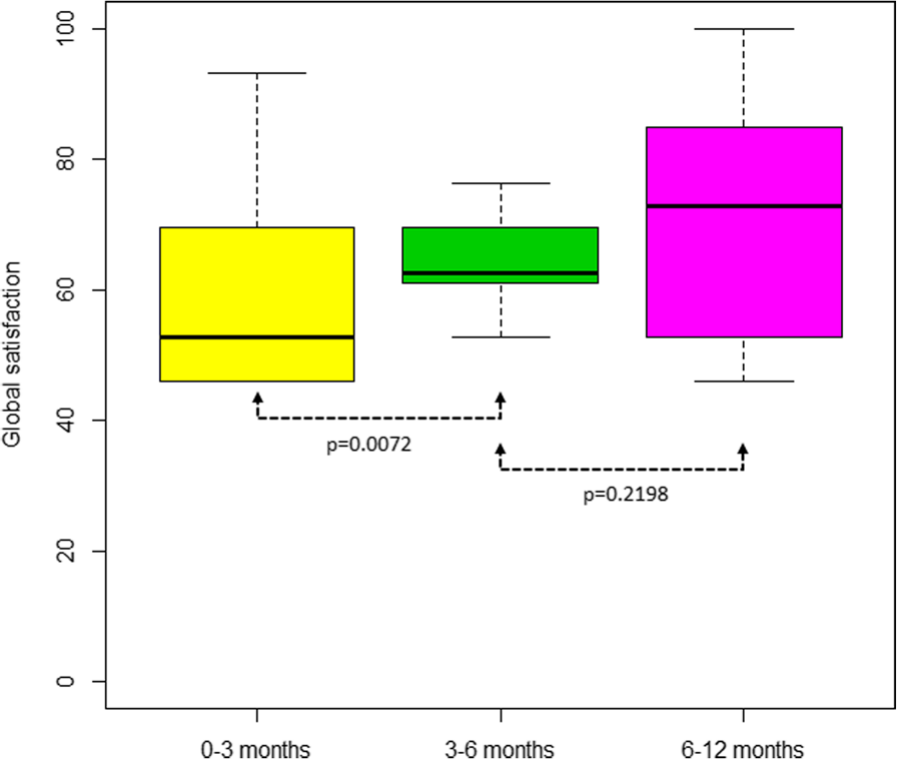
Box plot showing global satisfaction. The box plot shows the changes over time in global satisfaction regarding the self-administration of pnf C1-INH (Berinert®), as reported in the TSQM surveys (medians in bold)

Effectiveness median score of self-infusion was 67 (IQR: 50–78). The median score for the side effects was 100, meaning an extremely positive score. The number of attacks with side effects decreased over time, from 37% to 13%. The convenience of administration was consistent in the study period. Global satisfaction grew over time during the study period: this increase was statistically significant (*p* = 0.0072) between the first and the second quarter of drug use (Fig. 2).

### Admission to the emergency room/healthcare professional help

In the 3 years before enrollment, 100% of the attacks treated with Berinert® (129/year, *n* = 15) required admission to the ER or the help of a physician or a nurse, whereas during the study year only 8% (15/187) of the attacks treated with Berinert® required the ER or a healthcare professional.

## Discussion

This observational study assessed the feasibility and convenience of the home self-administration of C1-INH concentrate in patients with C1-INH-HAE and referring to the center for angioedema. According to the international guidelines on the management of acute attacks [6], self-administration should be offered as the best practice by a referral center [14, 21].

This strategy was successfully adopted also in other chronic conditions, e.g. hemophilia, and previous studies in patients with C1-INH-HAE reported that self-administration was safe. Self-infusion enables an early treatment, which in turn results in better outcomes.

Nevertheless, while training patients to perform self-infusions some barriers have to be considered. Tuong and collegues [22] reported that fear of injection or infection, lack of skills, interference of daily activities, and financial restraints are the main reasons why patients refuse to self-infuse.

In this survey, treatment was self-administered within 2 h and the complete resolution of the attacks occurred 6 h after treatment. In this real-world study the time to resolution is shorter than in the study IMPACT 2 [23], in which the median time to the complete resolution of symptoms was 15.5 h. In a previous study reporting real-world data on the treatment of acute attacks, the median total duration of the attacks treated with pnf C1-INH was 11.5 h [24]. This difference might depend on the fact that most attacks recorded were abdominal and their resolution is usually faster than peripheral attacks. In addition, Berinert® was promptly self-administered at home, thus resulting in shorter delay in treatment (delay in treatment is widely recognized as responsible of a poorer effectiveness of Berinert®).

Self-infusion was safe: no serious adverse events occurred. The number of attacks with adverse events decreased over time. This downtrend was likely due to an increased confidence in drug self-administration. The severity of the AEs was mostly mild-to-moderate. Severe swelling and severe pain were reported in only 18% and 8% of the attacks, respectively. However, among the attacks characterized by severe swelling, 32 out of 33 were attributed to the same patient. In addition, 13 out of 15 attacks giving rise to severe pain were attributed to one single patient.

The 1st quartile TSQM score on adverse effects was 100, meaning that at least 75% of the attacks had the maximum score in that domain. Therefore, in at least 75% of the attacks, the drug gave rise to no – or negligible – side effects.

Global satisfaction about the treatment increased over time during the study period, reaching statistical significance in the first 6 months. The percentage of patients reporting high levels of stress decreased over time, thus showing an increased confidence with the treatment and a higher ability in self-infusing the drug.

The increase in the quality of life recorded between the first and the third visit suggests that self-infusion was beneficial, although statistical significance was not reached. The perceived control of the illness improved over time and the analysis of the data shows that home treatment had a greater impact after 6 months of use, resulting in a median change in the quality of life score of 11 points (IQR: 4–14).

A limit of the study is the low sample size. Therefore, we recalculated the sample size for the change in the quality of life detected between baseline and the third visit at 6 months (mean = 6.4, SD = 13.5). Once set the significance level at 5% and the power at 80%, it would be necessary to enroll at least 67 patients to obtain statistically significant results, but in a real-world context it is difficult to recruit such a number of patients.

In the years before enrollment, the number of attacks treated with Berinert® was 129/year, while during the year of observation it was 189. This difference may be explained by the greater propensity by patients to treat the attacks with the specific and effective drug at home. Before the training, patients used to go to the ER or to search for healthcare professional help only to treat the most severe attacks, while at home they used non-specific drugs (symptomatic drugs or tranexamic acid). The data we collected emphasize that, after a self-infusion training, patients are more prone to treat the attacks with the specific drug, resulting in a better adherence to international guidelines.

Before the enrollment in the study, attacks treated with Berinert® requiring admission to ER/healthcare professional help were 100%. In the study year, only 8% of the attacks treated with Berinert® required admission to ER/healthcare professional help, thereby highlighting a reduction in the number of admissions to ER/healthcare professional help (*p* < 0.0001) and the related costs.

Only three out of 15 attacks that required admission to ER/healthcare professional help during the study period were treated in ER. One attack was laryngeal, while the other two attacks were abdominal. In these two abdominal attacks, the patients reported that symptoms were too severe to perform the self-infusion.

According to guidelines, patients self-treated attacks at home. Two out of three laryngeal attacks recorded during the study period were treated at home with prompt resolution of symptoms. Although these two attacks were successfully treated at home, medical evaluation for the risk for airways’ obstruction and consequent death for asphyxia is mandatory in case there is no prompt resolution of laryngeal symptoms.

## Conclusions

Self-administration of pnf C1-INH is safe and effective, increases patients’ confidence in the treatment and shows a trend in improving the quality of life. It reduces the need of admission to ER/healthcare professionals help for acute attacks and the related costs.

## Abbreviations

C1-INH: C1 esterase inhibitor
ENT: Ear, nose, and throat
ER: Emergency Room
HAE: Hereditary angioedema
HCP: Health care provider
ICU: Intensive care unit
IV: Intravenous
pnf: plasma-derived nanofiltered
QoL: Quality of life
TSQM: *Treatment satisfaction questionnaire for medication*
